# Supplementary material for: Usp7 regulates Hippo pathway through deubiquitinating the transcriptional coactivator Yorkie
Source: Nat Commun. 2019 Jan 24;10:411. doi: 10.1038/s41467-019-08334-7 (PMC6345853; doi:10.1038/s41467-019-08334-7)
Supplement: Supplementary file 1 — Supplementary Information [file 41467_2019_8334_MOESM1_ESM.pdf]

Supplementary Information

Usp7 regulates Hippo pathway through deubiquitinating the transcriptional coactivator Yorkie

Sun et al.

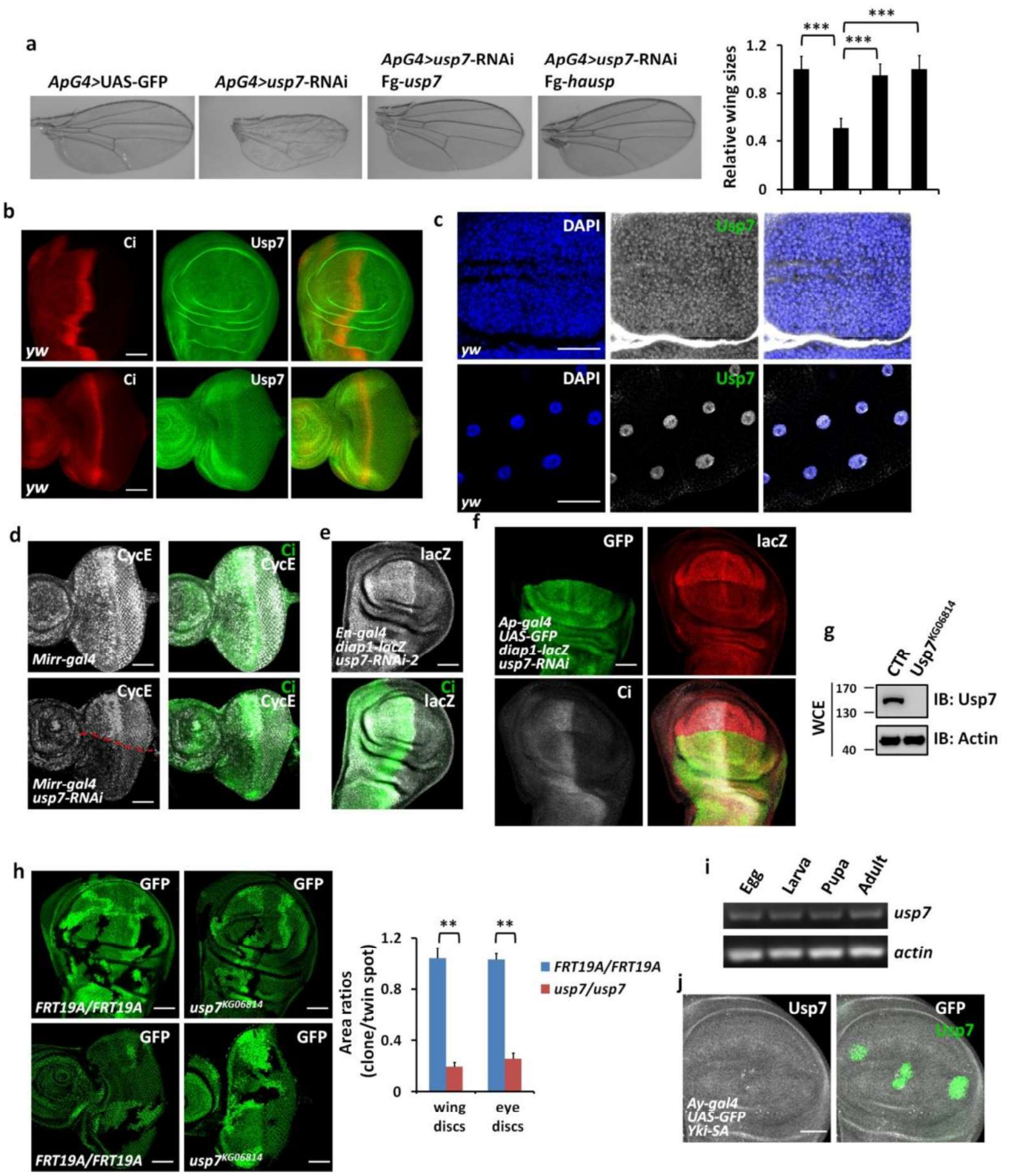

Supplementary Figure 1. Loss of *usp7* shows growth disadvantage. **a** Knockdown of *usp7* resulted in a small wing, which was rescued by overexpressing *usp7* or *hausp*. Quantification analyses were shown on the right. Data are means ± SEM. n=10 biological independent wings**.** ***P < 0.001 by student's *t*-test. **b** *usp7* evenly expressed in the wing and eye discs. **c** Usp7 protein mainly localized in the nucleus in the wing disc and salivary gland. DAPI marks the nucleus. **d** Eye discs of control (upper) or expressing *usp7* RNAi (down) were stained to show CycE (white) and Ci

(green). Of note, knockdown of *usp7* apparently decreased CycE level (below the red dashed line). **e** A wing disc expression *usp7* RNAi by *En-gal4* was stained to show Ci (green) and *diap1*-lacZ (white). **f** A wing disc expressing *usp7* RNAi by *ApG4* was stained to show GFP (green), *diap1*-lacZ (red) and Ci (white). Of note, knockdown of *usp7* equally decreased *diap1*-lacZ in the A and P compartments. **g** Immunoblots of control embryos and *usp7*^{KG06814} homozygote mutant embryos. *usp7*^{KG06814} was balanced with FM7.*Kr*-GFP and embryos loss of GFP signals were homozygotes. **h** *usp7* mutant clones showed growth defect. The control clones (FRT19A/FRT19A) and *usp7*^{KG06814} mutant clones were marked by loss of GFP. Quantification of the ratios of GFP$^{-/-}$ area/GFP$^{+/+}$ area (clones/twin spots) of wing discs and eye discs were shown on the right. Data are means ± SEM. n=10 biological independent discs. $^{**}P < 0.01$ by student's *t*-test. **i** Semi-quantative RT-PCR analysis was performed to examine the expression of *usp7* in distinct developmental stages of *Drosophila*. Of note, *usp7* steadily expresses from egg to adult. *actin* acts as a loading control. **j** A wing disc overexpressing *yki-SA* was stained to show GFP (green) and Usp7 (white). Scale bars: 50μm for all images.

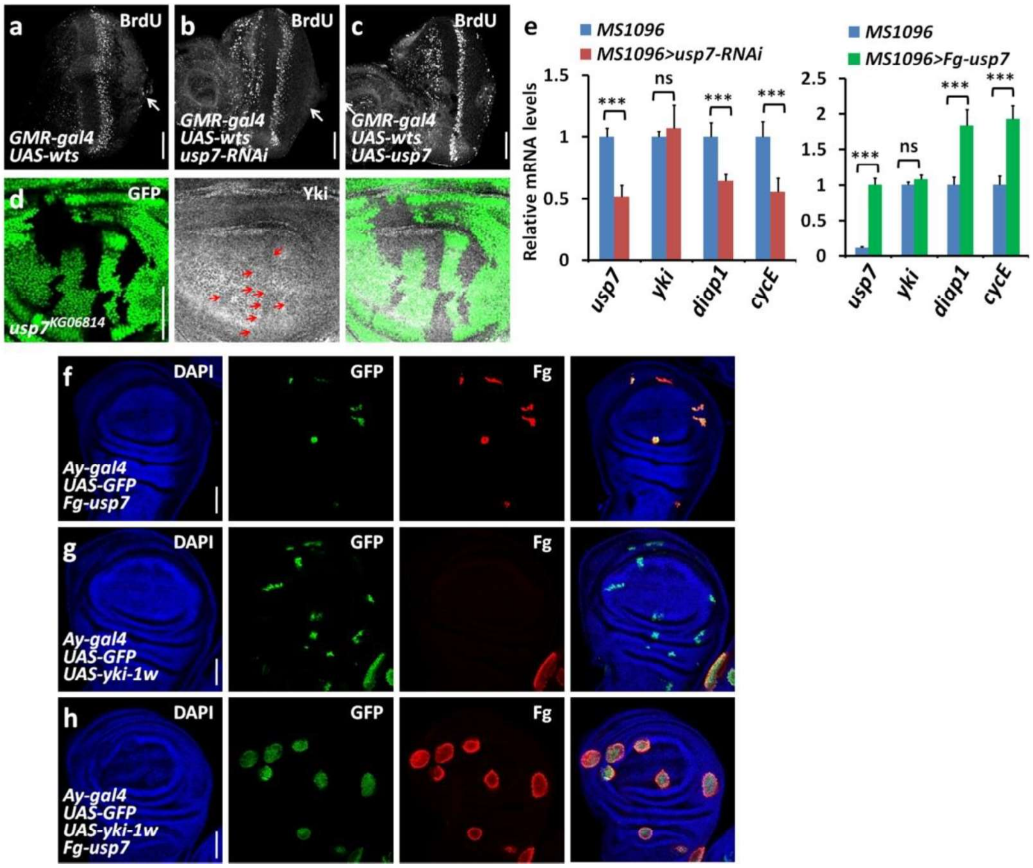

Supplementary Figure 2. Usp7 regulates Hippo pathway upstream of Yki. **a** A control eye disc was stained to show BrdU incorporation. Of note, some cells showed BrdU positive (arrow). **b-c** Knockdown of *usp7* decreased, while overexpression of *usp7* increased BrdU incorporation in the posterior regions (arrows). **d** A wing disc carrying *usp7*^{KG06814} clones were stained to show the expression of GFP (green) and Yki (white). Of note, *usp7* mutation resulted in Yki expression decreased (arrows). **e** Relative mRNA levels of *usp7*, *yki*, *diap1* and *cycE* from indicated wing discs were revealed by real-time PCR. Data are means ± SEM. n=3 biological independent experiments. ns, not significant, ***P < 0.001 by student's *t*-test. **f** A wing disc expressing Fg-*usp7* by *Ay-gal4* (*act>y+>gal4*) was stained to show DAPI (blue), GFP

(green) and Fg (red). GFP marks the Fg-*usp7*-overexpressing clones. **g** A wing disc expressing *yki-1w* was stained to show DAPI (blue), GFP (green) and Fg (red). **h** Simultaneous expression of *yki-1w* and *usp7* produced larger and rounder clones. Scale bars: 50μm for all images.

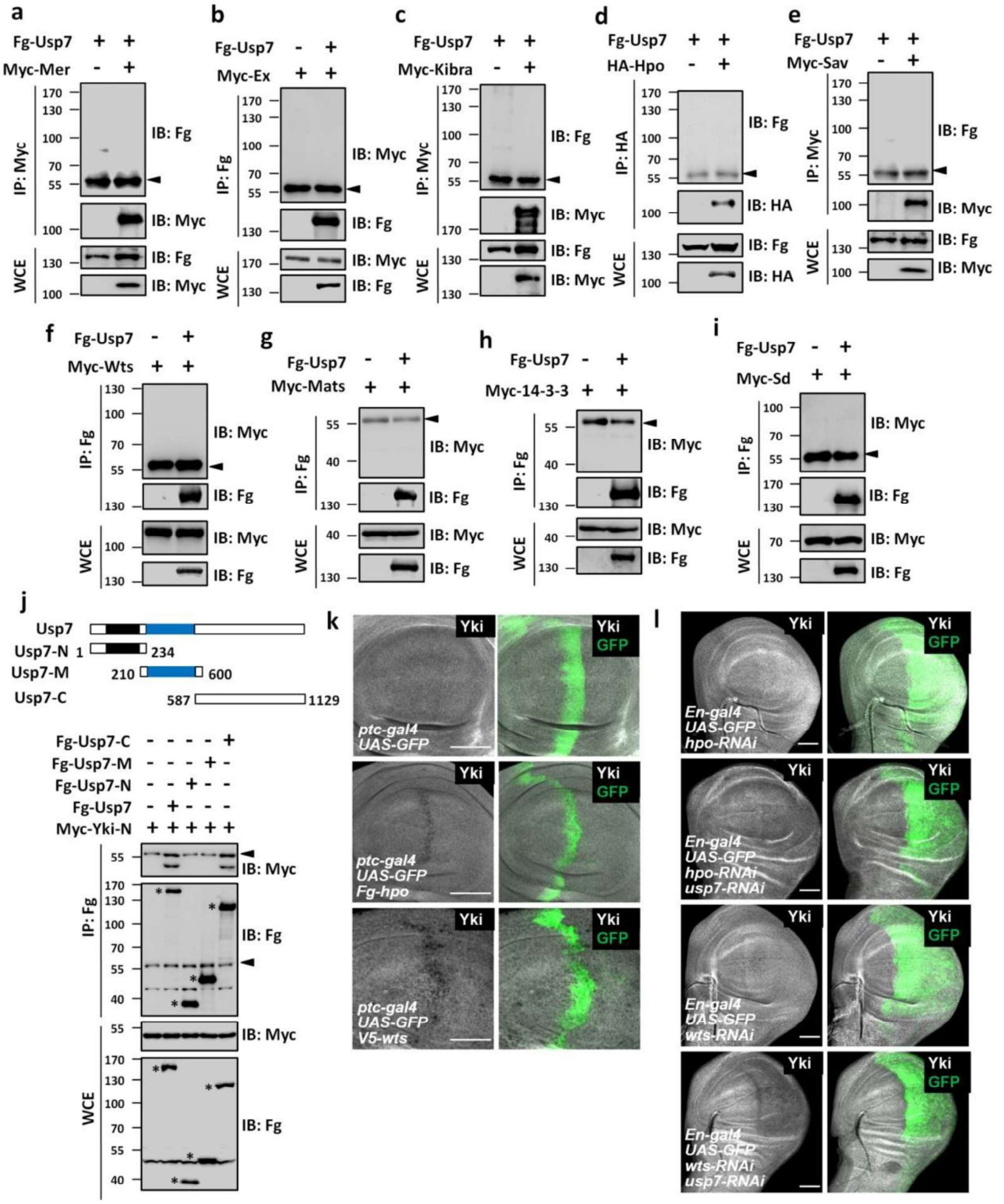

Supplementary Figure 3. Usp7 does not bind many components of Hippo signaling. **a** Immunoblots of immunoprecipitates (top two panels) or lysates (bottom two panels)

from S2 cells expressing indicated proteins. Usp7 did not show any interaction with Mer. **b** Usp7 did not bind Ex. **c** Usp7 did not bind Kibra. **d** Usp7 did not bind Hpo. **e** Usp7 did not bind Sav. **f** Usp7 did not bind Wts. **g** Usp7 did not bind Mats. **h** Usp7 did not bind 14-3-3. **i** Usp7 did not bind Sd. **j** Myc-Yki-N interacted with the C-terminal region of Usp7. Asterisks marked the truncated Usp7 proteins. All above, the arrowhead indicated heavy IgG. **k** Overexpression of *hpo* or *wts* decreased Yki protein, which was rescued by simultaneous *usp7* overexpression. **l** Knockdown of *hpo* or wts elevated Yki protein, which was restored by *usp7* knockdown. Scale bars: 50μm for all images.

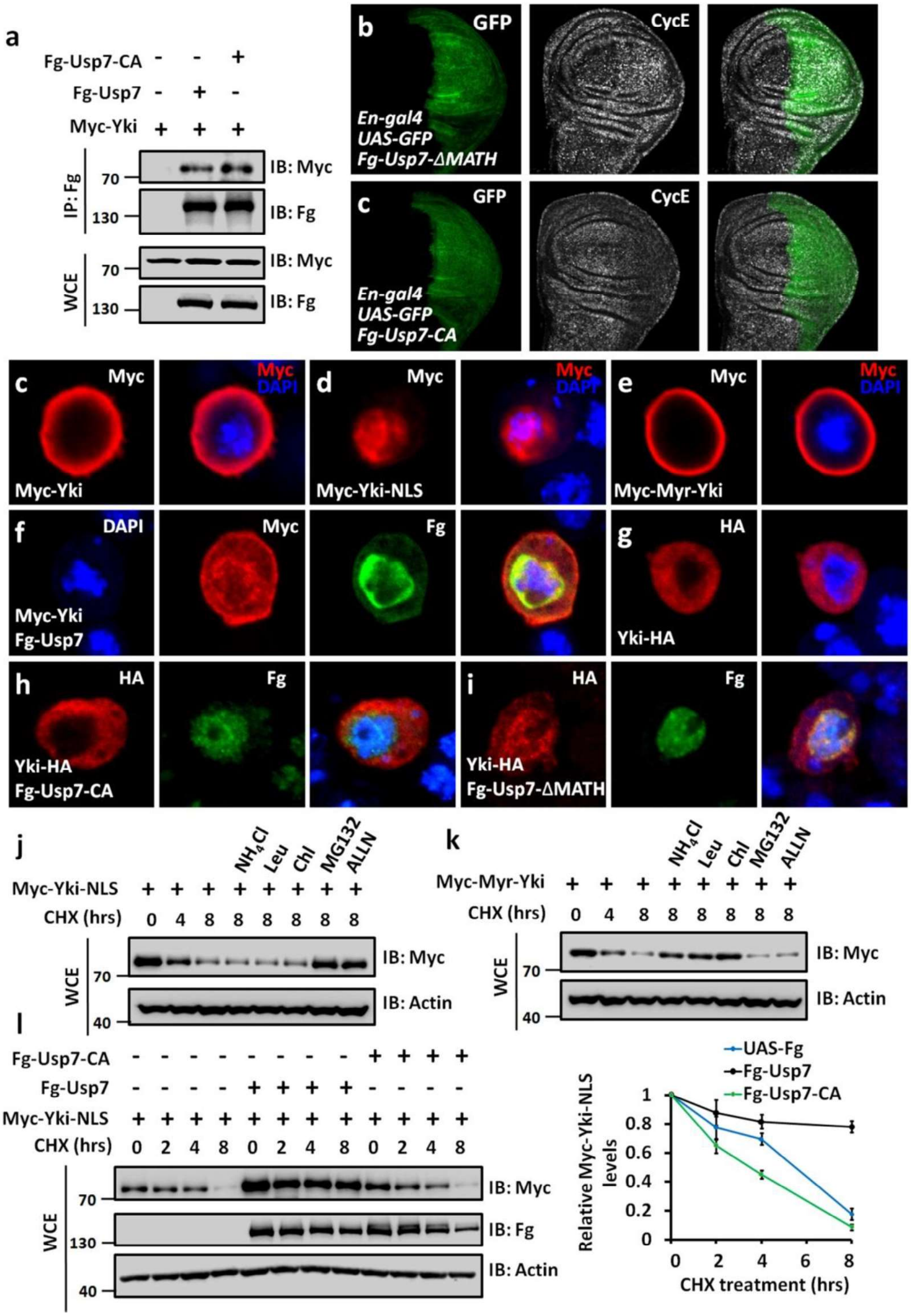

Supplementary Figure 4. Usp7 stabilizes the nuclear Yki via its deubiquitinase activity. **a** Immunoblots of immunoprecipitates (top two panels) or lysates (bottom two panels) from S2 cells expressing indicated proteins. Both Usp7 and Usp7-CA

could bind Yki. **b** A wing disc expressing *usp7-ΔMATH* was stained to show GFP (green) and CycE (white). Overexpression of *usp7-ΔMATH* caused CycE elevation. **c** A wing disc expressing *usp7-CA* was stained to show GFP (green) and CycE (white). Overexpression of *usp7-CA* decreased CycE level. **c** A S2 cell transfecting Myc-Yki was stained to show Myc (red) and DAPI (blue). **d** Myc-Yki-NLS exclusively localized in the nucleus. **e** Myc-Myr-Yki only localized in the cytoplasm. **f** S2 cells co-expressing Myc-Yki and Fg-Usp7 proteins were stained to show DAPI (blue), Myc (red) and Fg (green). Notably, Usp7 promoted Yki nuclear accumulation. **g** Yki-HA protein mainly localized in the cytoplasm. **h** Usp7-CA failed to promote Yki protein nuclear accumulation. **i** Usp7-ΔMATH could promote Yki protein nuclear accumulation. **j** S2 cells transfected with Myc-Yki-NLS were treated with CHX plus distinct inhibitors for indicated intervals. NH_4Cl, leupeptin (Leu) and chloroquine (Chl) were lysosome inhibitors. MG132 and ALLN were proteasome inhibitors. Of note, MG132 and ALLN could hamper Myc-Yki-NLS degradation. **k** S2 cells transfected with Myc-Myr-Yki were treated with CHX plus distinct inhibitors for indicated intervals. Notably, Myr-Yki was degraded by lysosome. **l** Immunoblots of lysates from S2 cells expressing indicated constructs and treated with CHX for indicated intervals. Quantification analyses were shown on right of the autoradiogram. The results were presented as means ± SEM of values from three independent experiments. Above all, Actin acts as a loading control.

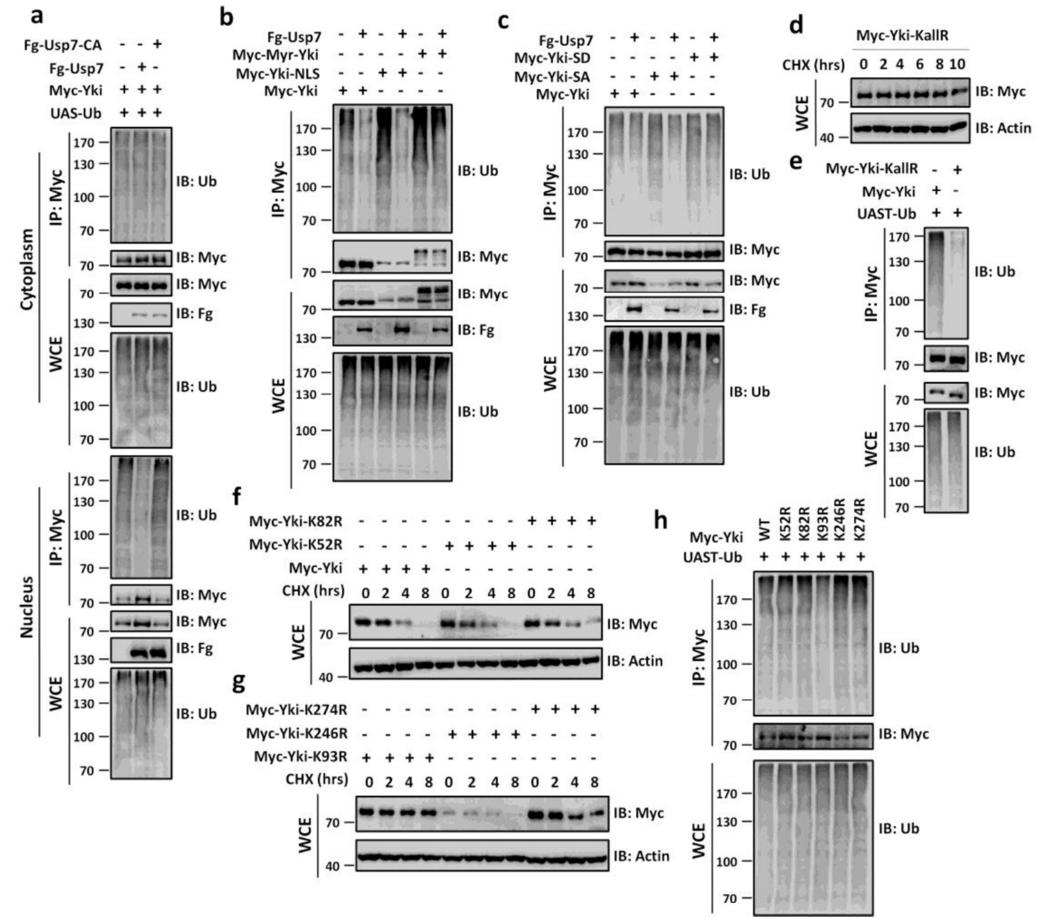

Supplementary Figure 5. Usp7 deubiquitinates the nuclear Yki protein. **a** Usp7 mainly deubiquitinated nuclear Yki protein. S2 cells transfected by indicated constructs were analyzed by subcellular fractionation. Before cell harvesting, cells were treated by NH_4Cl and MG132 for 4hrs. **b** Usp7 effectively deubiquitinated Yki-NLS, not Myr-Yki. Before cell harvesting, cells were treated by NH_4Cl and MG132 for 4hrs. **c** Usp7 robustly removed ubiquitin chains from Yki and Yki-SA, not Yki-SD. Before cell harvesting, cells were treated by NH_4Cl and MG132 for 4hrs. **d** S2 cell transfected by Myc-Yki-KallR were treated by CHX for indicated intervals. Of note, Myc-Yki-KallR was stable. Actin acts as a loading control. **e** Myc-Yki-KallR failed to be ubiquitinated. Before cell harvesting, cells were treated by NH_4Cl and

MG132 for 4hrs. **f-g** S2 cells transfected with indicated Yki mutant constructs were treated by CHX for indicated intervals. Notably, mutation of K93 hampered Yki degradation. Actin acts as a loading control. **h** Mutation of K93 on Yki inhibited its ubiquitination. Before cell harvesting, cells were treated by NH_4Cl and MG132 for 4hrs.

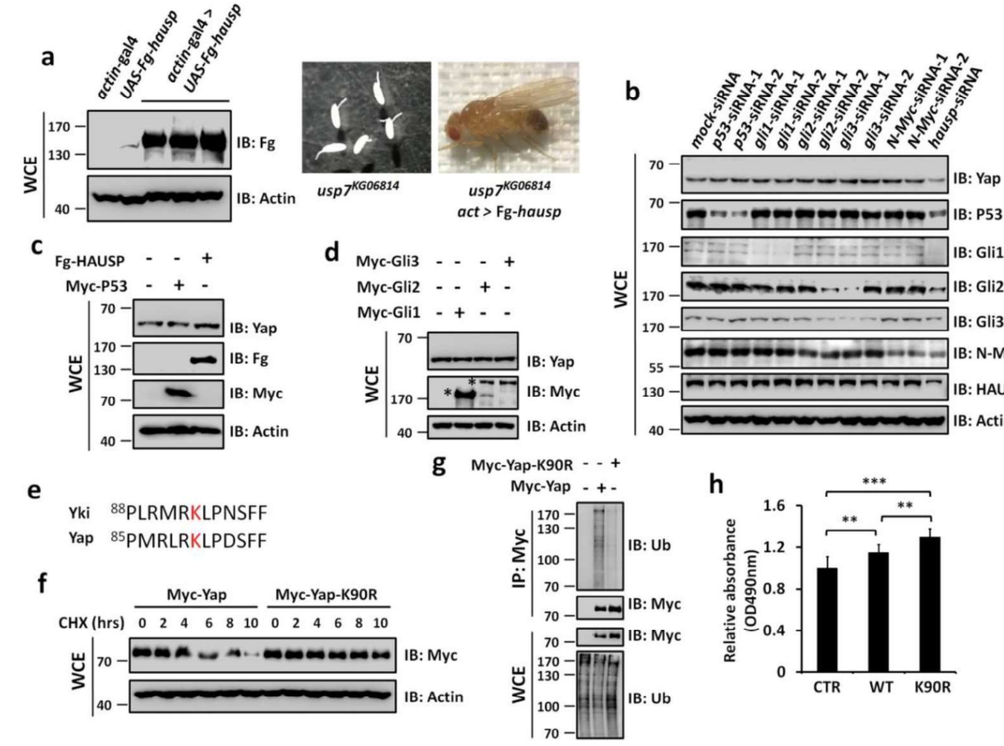

Supplementary Figure 6. HAUSP plays a conserved role to deubiquitinate Yap. **a** Overexpression of *hausp* could rescue the lethality by *usp7* mutation in *Drosophila*. Flies from indicated genotypes were lysed by cell lysis buffer and then underwent IB assay. Actin acts as a loading control. Of note, *usp7*^{KG06814} homozygotes (GFP negative embryos from *usp7*^{KG06814}/FM7. *Kr*-GFP) were embryonic lethality, which were restored by *hausp* expression. **b** Immunoblots of Huh7 cells transfected with indicated siRNAs. Of note, knockdown of HAUSP targets did not decrease Yap

protein level. Actin acts as a loading control. **c-d** Immunoblots of 293T cells transfected by indicated constructs. Overexpression HAUSP target genes did not show any effect on Yap protein. Actin acts as a loading control. **e** K93 on Yki protein was conserved in human Yap. **f** 293T cells transfected with Myc-Yap or Myc-Yap-K90R treated with CHX for indicated intervals. Notably, Yap-K90R showed slower degradation than wild type Yap. Actin acts as a loading control. **g** Ubiquitination assays of 293T cells transfected with indicated constructs. Yap-K90R showed lower ubiquitination than wild type Yap. Before cell harvesting, cells were treated by NH_4Cl plus MG132 for 4hrs. **h** MTT assays of Huh7 cells transfected with wild type Yap or Yap-K90R. Notably, Yap-K90R showed higher efficiency in promoting cell proliferation than wild type Yap. Data are means ± SEM. n=3 biological independent experiments. ns, not significant, $^{**}P < 0.01$ and $^{***}P < 0.001$ by student's *t*-test.

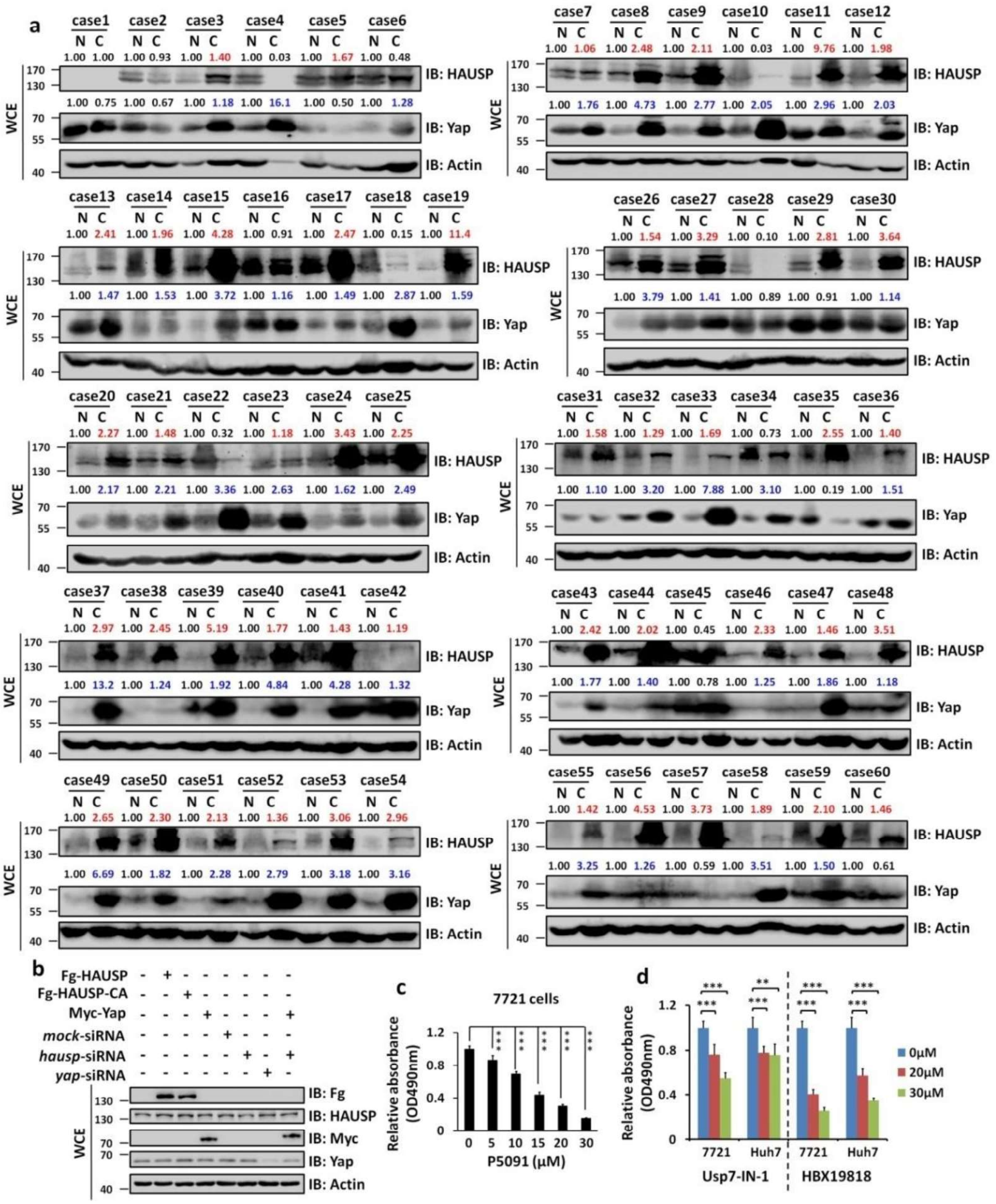

Supplementary Figure 7. HCC samples show increased HAUSP and Yap. **a** Immunoblots of HAUSP and Yap protein levels in 60 paired of HCC tissues and nontumor tissues (N, paratumor normal sample; C, HCC sample). Of note, most HCC samples showed simultaneously increased HAUSP and Yap proteins. We measured the protein bands using Image J, and normalized to Actin. The numbers on figures represented the relative intensities of bands to corresponding nontumor bands. The red

numbers marked HAUSP increased cases, while the blue numbers marked Yap increased cases. **b** Immunoblots of lysates from Huh7 cells expressing indicated constructs or siRNAs. Of note, *hausp*-siRNA and *yap*-siRNA could effectively silence the endogenous genes. Actin acts as a loading control. **c** The proliferation of 7721 cells treated with P5091 at indicated concentrations for 24hrs. **d** The proliferation of 7721 cells and Huh7 cells treated by Usp7-IN-1 or HBX19818 at indicated concentrations for 24hrs. Above all data are means ± SEM. n=3 biological independent experiments. $^{***}P < 0.001$ by student's *t*-test.

Supplementary Figure 8

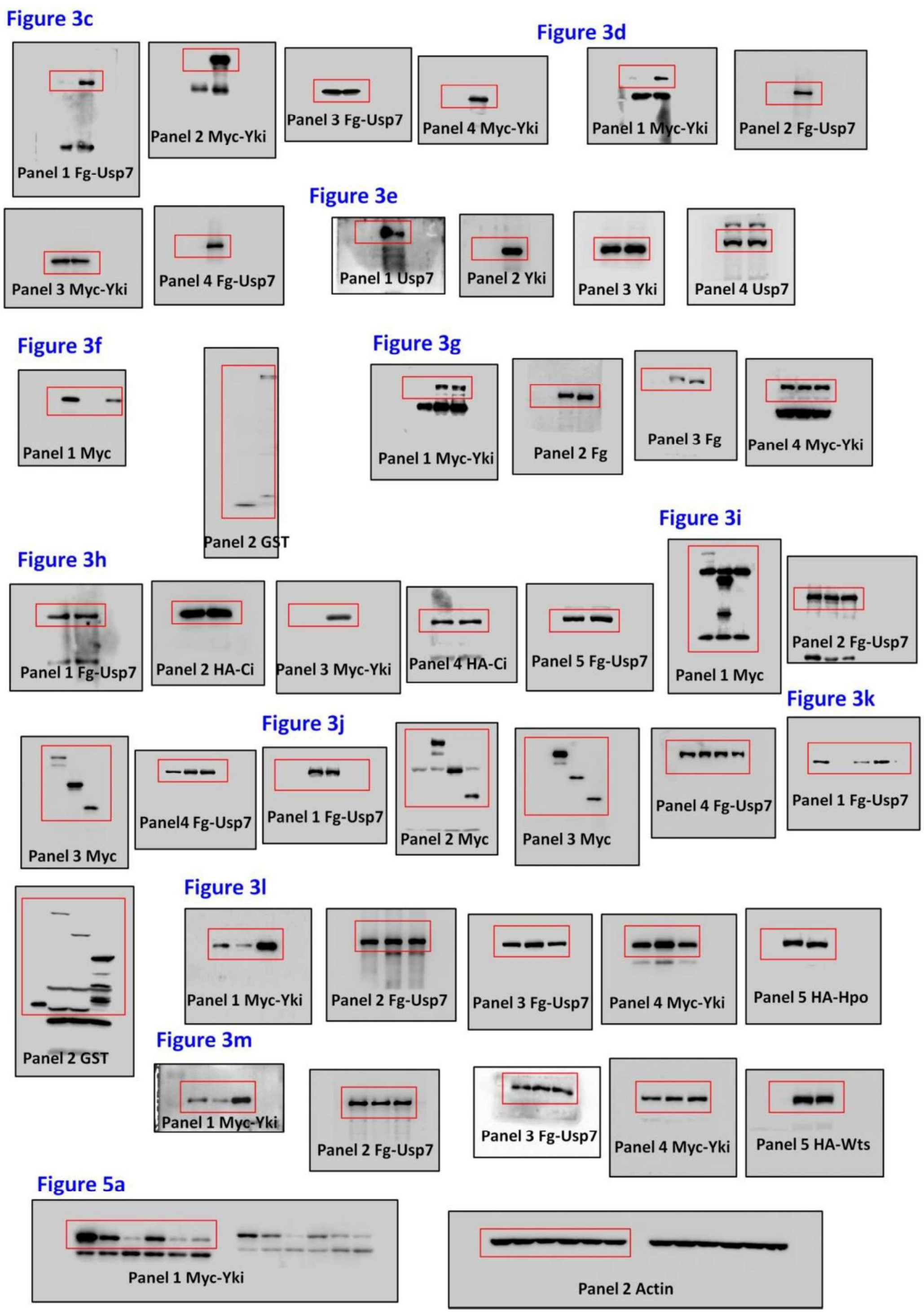

Original immunoblotting figures

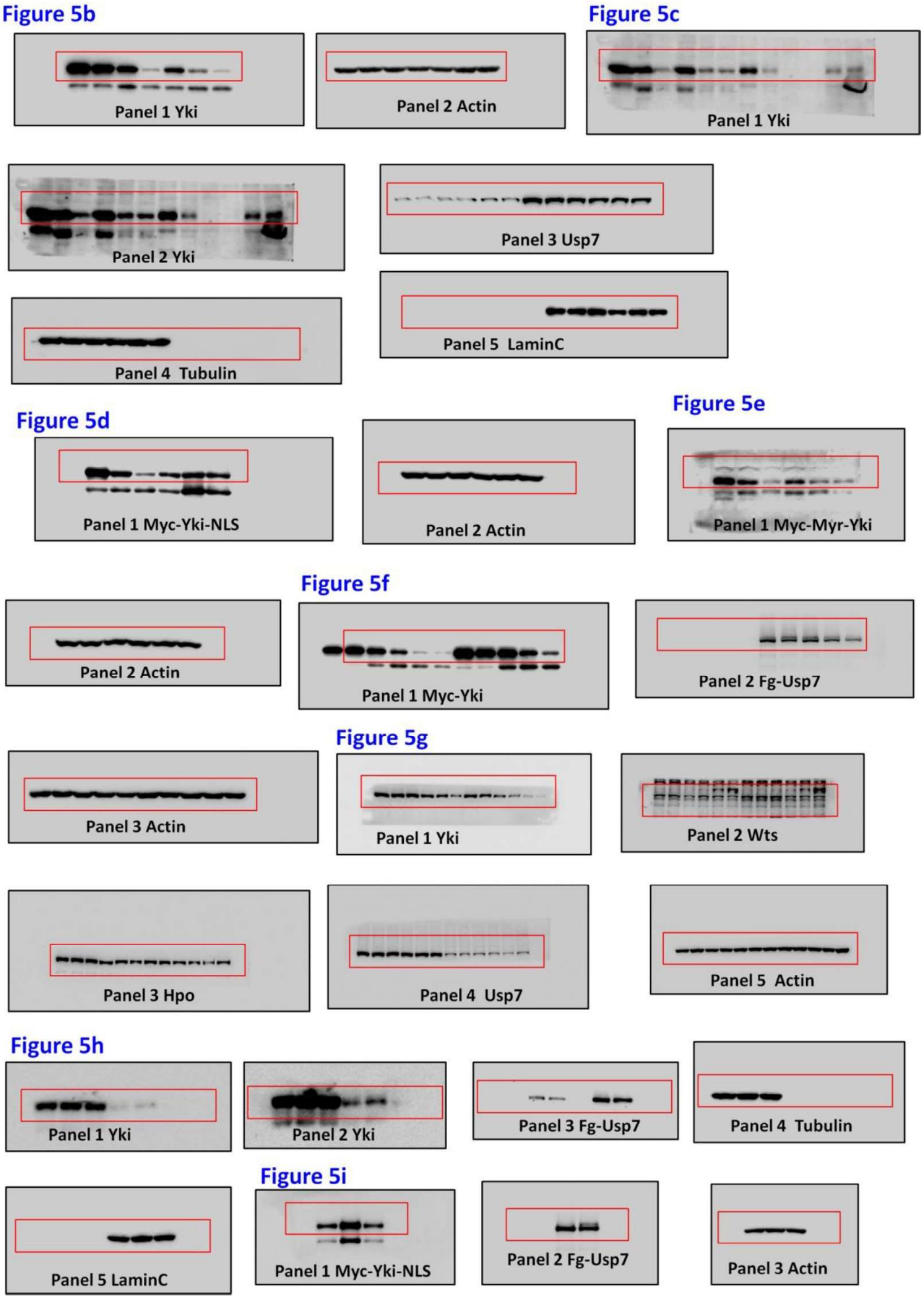

Original immunoblotting figures

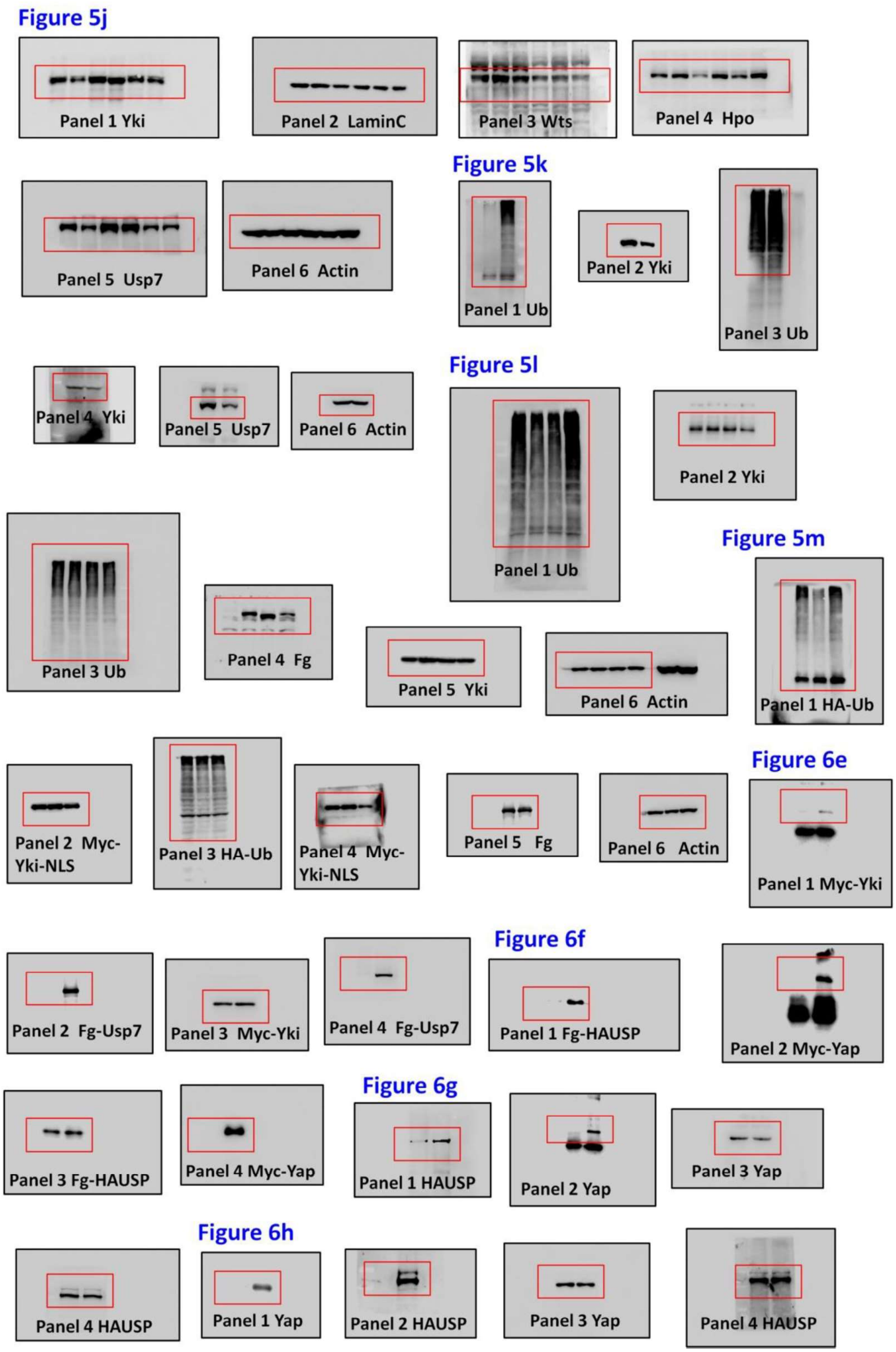

Original immunoblotting figures

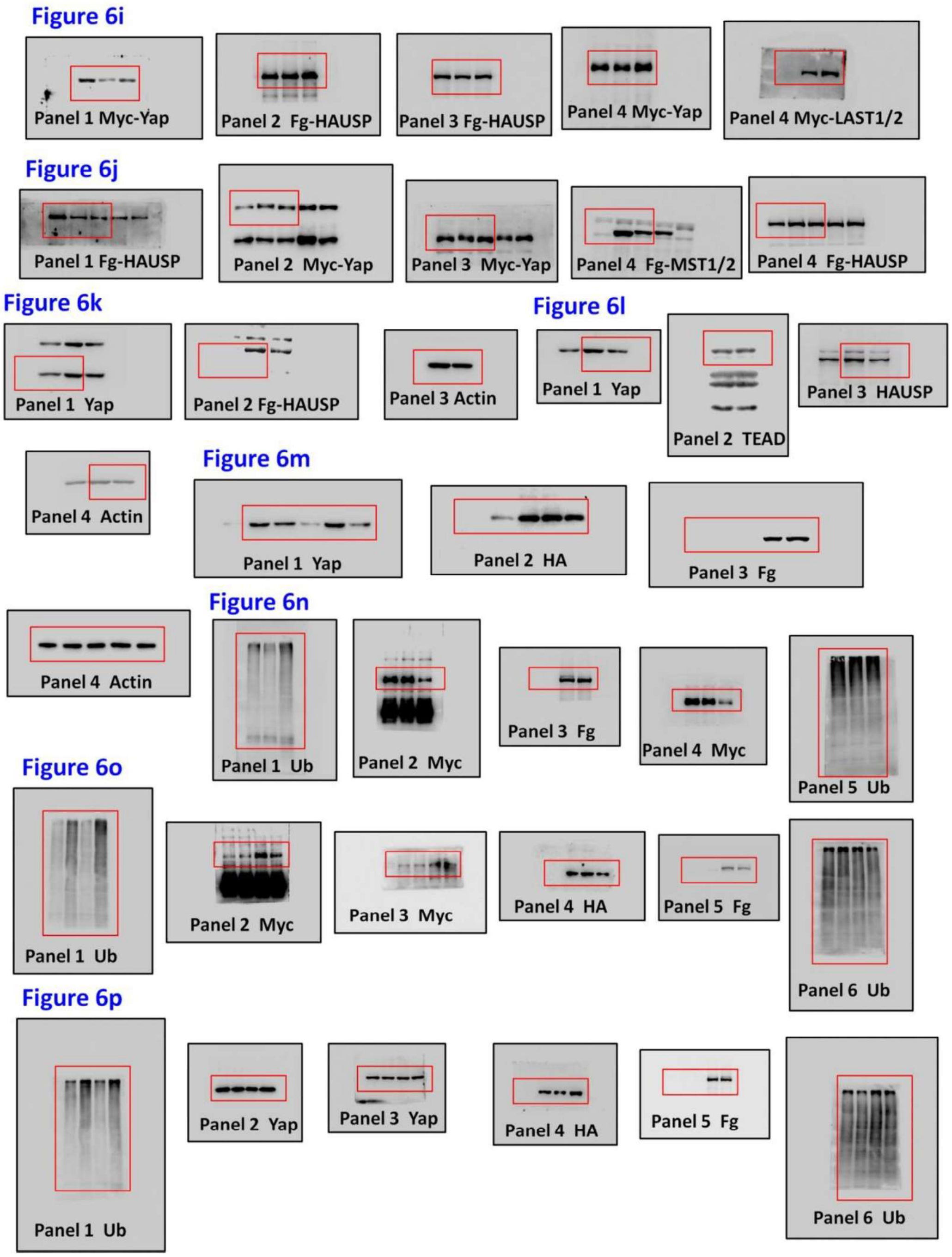

Original immunoblotting figures

Figure 7h

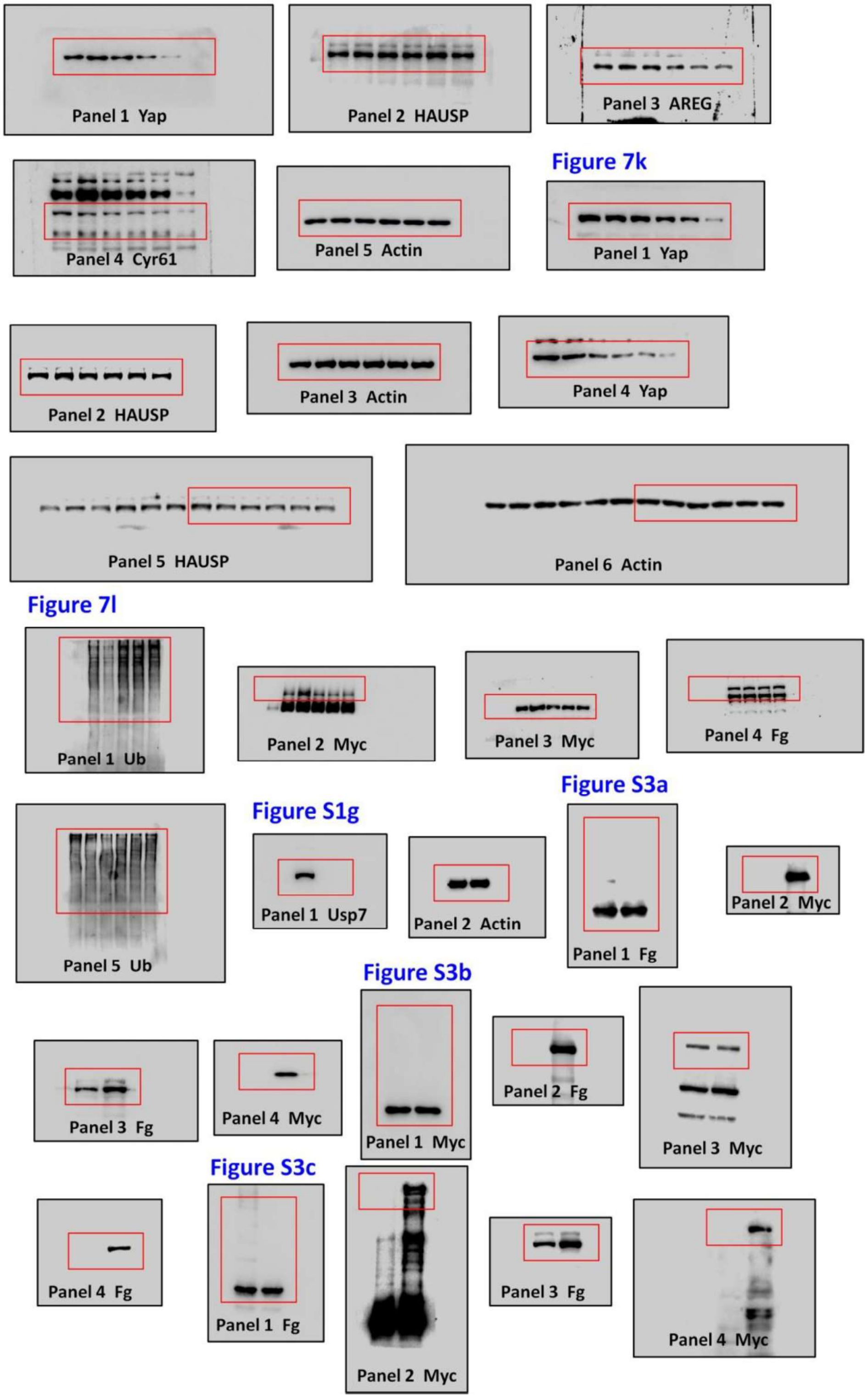

Original immunoblotting figures

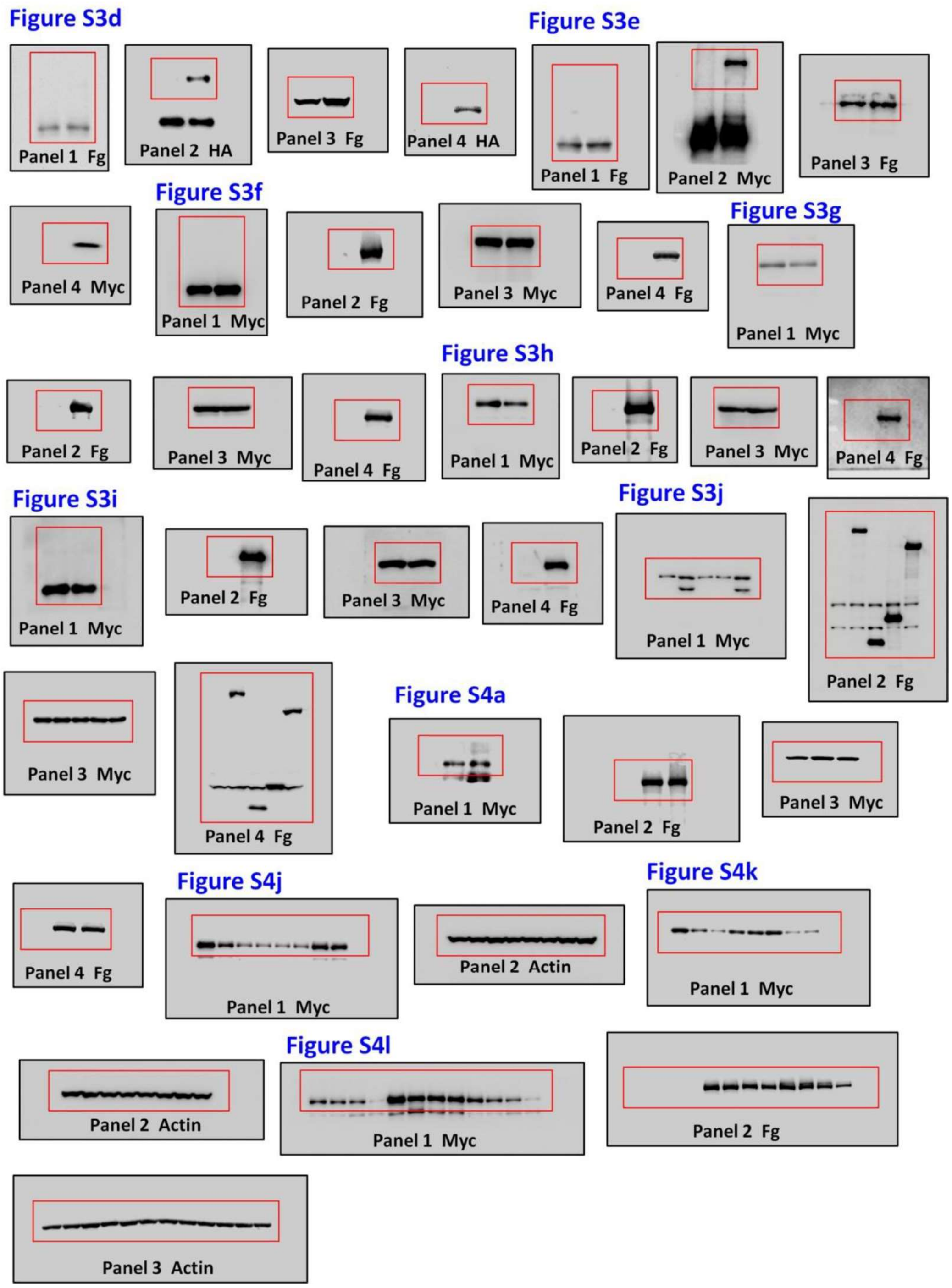

Original immunoblotting figures

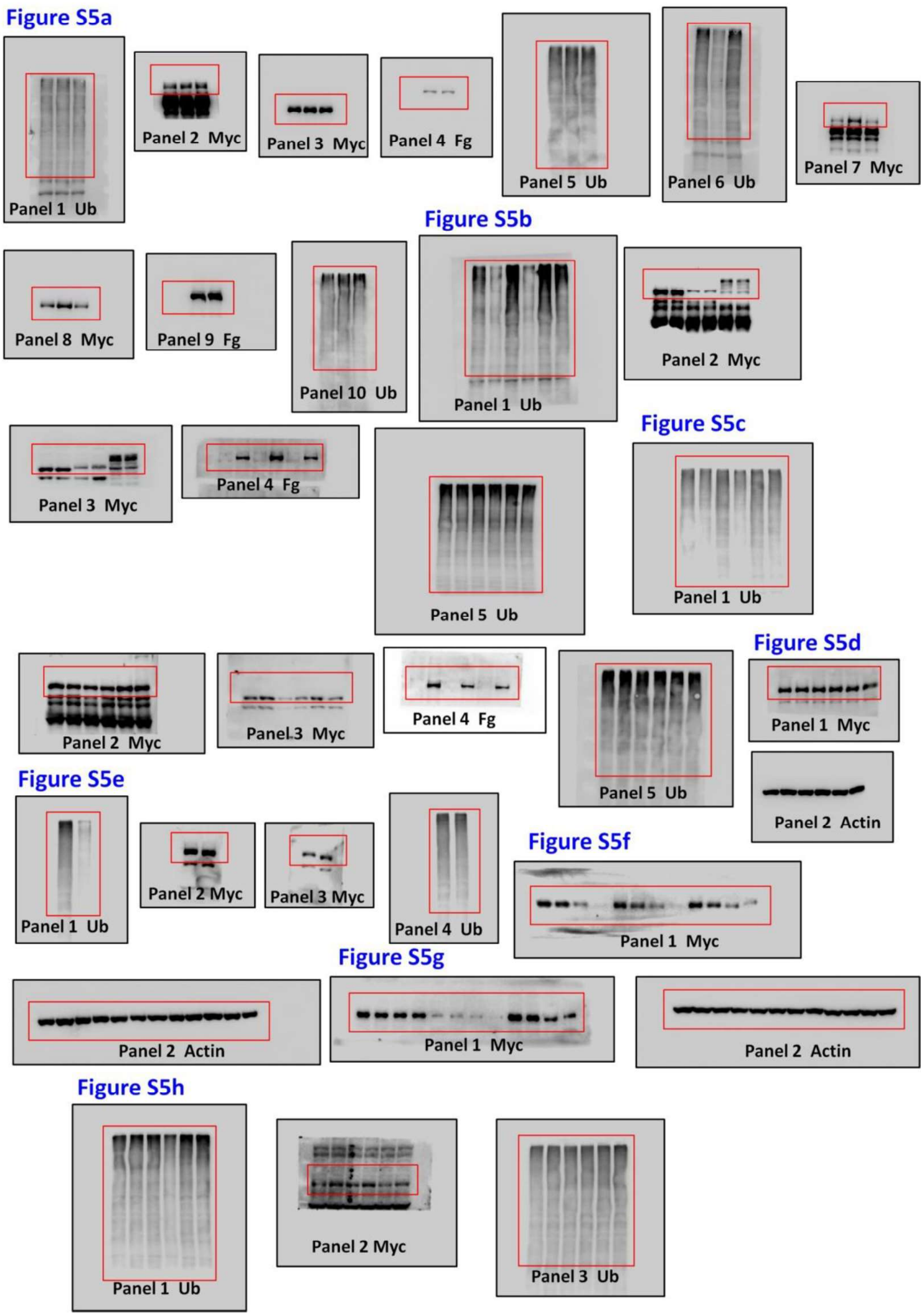

Original immunoblotting figures

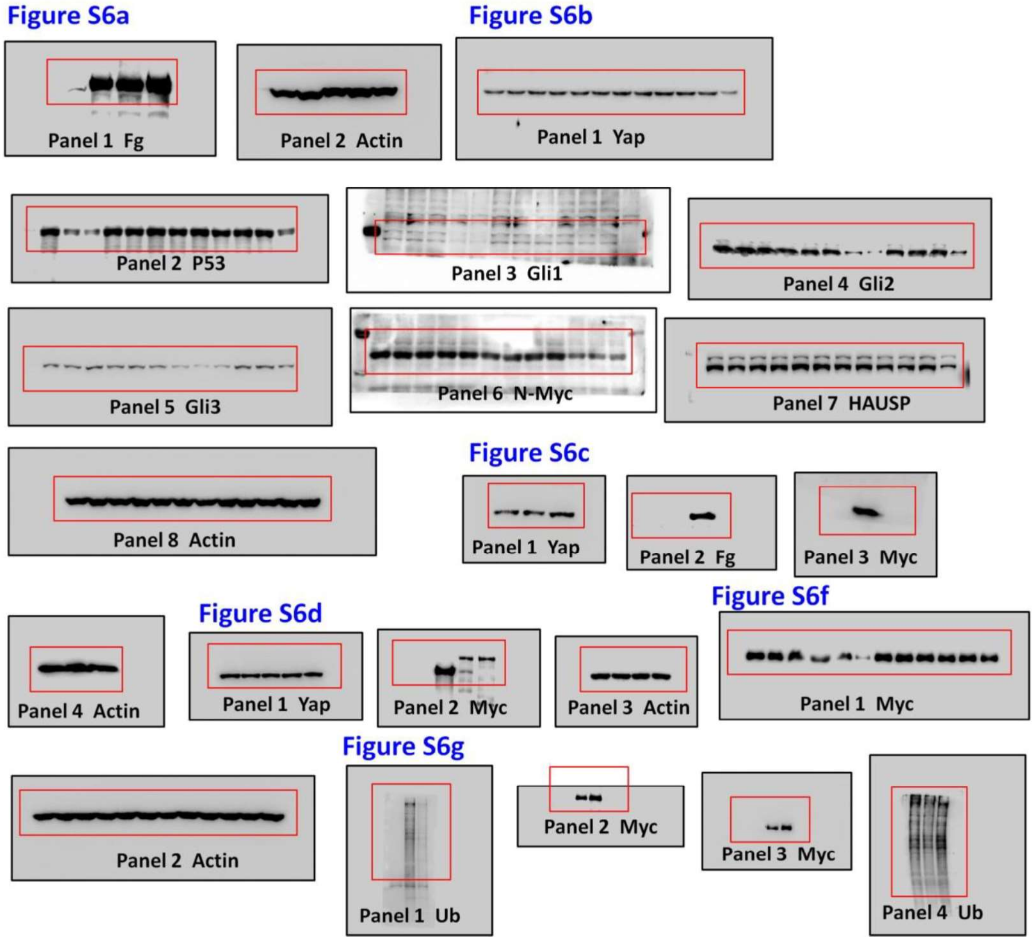

Original immunoblotting figures
